# Therapeutic Potential of Extracts from *Macaranga tanarius* (MTE) in Diabetic Nephropathy

**DOI:** 10.3390/plants12030656

**Published:** 2023-02-02

**Authors:** Yung-Chien Hsu, Cheng-Chih Chang, Ching-Chuan Hsieh, Ya-Hsueh Shih, Hsiu-Ching Chang, Chun-Liang Lin

**Affiliations:** 1 Department of Nephrology, Chang Gung Memorial Hospital, Chiayi 613, Taiwan; 2 Kidney and Diabetic Complications Research Team (KDCRT), Chang Gung Memorial Hospital, Chiayi 613, Taiwan; 3 Division of General Surgery, Chang Gung Memorial Hospital, Chiayi 613, Taiwan; 4 School of Traditional Chinese Medicine, College of Medicine, Chang Gung University, Taoyuan 333, Taiwan; 5 Kidney Research Center, Chang Gung Memorial Hospital, Taipei 105, Taiwan; 6 Center for Shockwave Medicine and Tissue Engineering, Chang Gung Memorial Hospital, Kaohsiung 833, Taiwan

**Keywords:** diabetes nephropathy, fibrosis, *Macaranga tanarius* extract (MTE)

## Abstract

Diabetic nephropathy is a complication of diabetes that leads to end-stage kidney disease and is a major health burden worldwide. Prenylflavonoid compounds extracted from *Macaranga tanarius* (MTE) exhibit anti-inflammation, anti-oxidant, and anti-bacterial properties. However, the effects of these compounds on diabetic nephropathy remain unclear. The effects of MTE on diabetic nephropathy were investigated in vitro by using mouse renal mesangial cells and in vivo by using a db/db knockout mouse model. No overt alteration in proliferation was observed in mouse renal mesangial cells treated with 0–1 μg/mL MTE. Western blot analysis indicated that MTE dose-dependently attenuated the expression of fibronectin, α-smooth muscle actin, and collagen IV. Administration of MTE ameliorated renal albumin loss in db/db mice. Immunohistochemical staining revealed that MTE mitigated diabetes-induced fibronectin and collagen IV expression. Periodic acid–Schiff (PAS) and trichrome staining also showed that administration of MTE reduced the renal fibrosis phenomenon. MTE significantly ameliorated diabetes-induced nephropathy.

## 1. Introduction

Diabetes mellitus (DM), one of the major metabolic disorders, has a high prevalence (approximately 10.5% of adults in 2021) worldwide [1]. The incidence of DM has increased every year and it is a significant health burden [1]. Hyperglycemia accumulates reactive oxygen species and promotes complications of DM such as cardiovascular diseases, retinopathy, and nephropathy. Diabetic nephropathy (DN) could lead to end-stage kidney disease and then cause renal dysfunction [2]. Renal fibrosis caused by the massive accumulation of extracellular matrix (ECM) including fibronectin and collagen IV in the glomerulus is the major characteristic of DN [3]. A limited number of drugs have been shown to block the progression of DN. Developing agents to rescue or attenuate the progression of DN with fewer side effects and costs is an urgent issue.

Natural compounds derived from herbs have received much interest because of their anti-oxidant, anti-inflammation, and anti-lipidemia properties. These compounds can ameliorate renal fibrosis induced by DN [4,5]. In one study, administration of Zingiber officinale extracts prevented streptozotocin (STZ)-induced renal cell apoptosis through its anti-oxidant and anti-inflammation abilities [6]. Cinnamon and its procyanidin-B2 (MTEB2)-enriched fraction attenuated urine albumin and creatinine levels in diabetic mice. In db/db mice, administration of curcumin significantly blocked the NLR family pyrin domain containing three inflammasome signaling (NLRP3) and reduce DN progression [7]. Polyphenols from Physalis peruviana fruits significantly reduced serum creatinine levels and attenuated positive periodic acid–Schiff (PAS) staining signals in STZ-induced diabetic mice [8]. Chtourou et al. demonstrated that STZ induced neutrophil infiltration and hypertrophied podocyte, whereas administration of a mixture of naringin, chlorogenic acid, and quercetin significantly reversed the phenomenon [9]. Administration of Shenkang pills, a traditional Chinese medicinal formula, evidently downregulated the activities of aurora kinase B and Ras homolog family member A (RhoA) and ameliorated renal podocyte dysfunction [10]. Xu et al. demonstrated that extracts from *Ranunculus ternatus* Thunb (RTT) significantly suppressed fibronectin, a-smooth muscle actin (a-SMA) and vimentin in STZ-induced diabetic mice. The expression of inflammation factors such as NF-kB and tumor necrosis factor-a was also mitigated by RTT [11].

*Macaranga tanarius* (L.) Mull.-Arg. (Euphorbiaceae) is a pioneer tree that grows first ahead of other plants on impoverished soil [12]. It grows throughout eastern and southern Asia. The root and bark of *Macaranga tanarius* are used as a folk medicine in Taiwan for treating hemoptysis and dysentery, respectively [12]. *M. tanarius* contains various chemical components such as prenylflavonoids [12], lignan glucosides [13], and terpenes [12] and exhibits a broad spectrum of biological activities. Chen et al. demonstrated that an extract from Macaranga exerted strong free radical scavenging activity [14]. Prenylflavonoids extracted from *Macaranga tanarius* promoted the differentiation of dental pulp stem cells via mitogen-activated protein kinase and protein kinase B pathways [15]. Treatment of L6 myotube cells with prenylflavonoids isolated from *M. tanarius* fruits dose-dependently stimulated AMP-dependent protein kinase activity and glucose transporter 1 (GULT1) expression and triggered glucose uptake [16]. Lee et al. showed that extracts from *Macaranga tanarius* exhibited anti-bacterial and anti-fugal properties [17]. Nymphaeol-C, one chemical isolated from *Macaranga tanarius*, significantly repressed fibroblast growth factor 8 expression and its downstream signals through downregulation of β-catenin [18]. Recently, the anti-diabetes properties of prenylflavonoid have received great attention. Two prenylflavonoids from Epimedii Herba prevented advanced glycation end-product formation and may exhibit an anti-diabetes ability [19]. Zhao et al. demonstrated that prenylflavonoid glycosides extracted from Epimedium koreanum attenuated lung fibrosis formation [20]. Xanthohumol, a prenylated flavonoid, enhanced the activity of nuclear factor erythroid 2-related factor 2 (Nrf2) and subsequently facilitated wound healing in diabetic mice [21]. Administration of 10 mg/kg O-prenylated flavonoid purified from Melicope lunu-ankenda leaves for 20 days evidently attenuated blood glucose levels in streptozotocin (STZ)-induced diabetic mice [22]. No study has reported the effects of prenylflavonoid compounds (MTE) derived from *Macaranga tanarius* on diabetic complications. In the present study, we investigated the effects of MTE on DN.

## 2. Results

### 2.1. Cytotoxicity Effect of MTE on Mouse Renal Mesangial Cells

**Figure 1:**
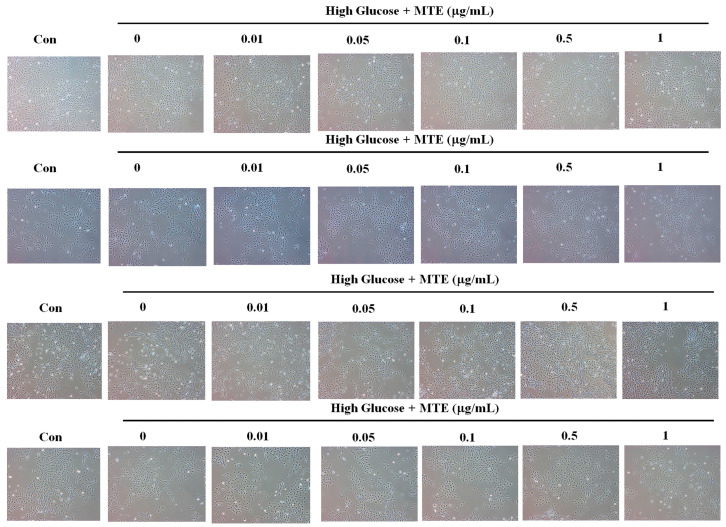
Cytotoxicity of prenylflavanone compounds of mouse renal mesangial cells. The mouse renal mesangial cells were treated with the indicated concentrations of MTE for 12, 24, 48, and 72 h (from upper to lower). The cell morphology was pictured by light contract microscope.

To evaluate the cytotoxicity of MTE on mouse renal mesangial cells, we treated the cells with different concentrations of MTE for 12, 24, 48, or 72 h. Figure 1 shows no overt alteration in the mouse renal mesangial cells in the presence of high glucose combined with 0, 0.01, 0.05, 0.1, 0.5, and 1 μg/mL MTE for the indicated times.

**Figure 2:**
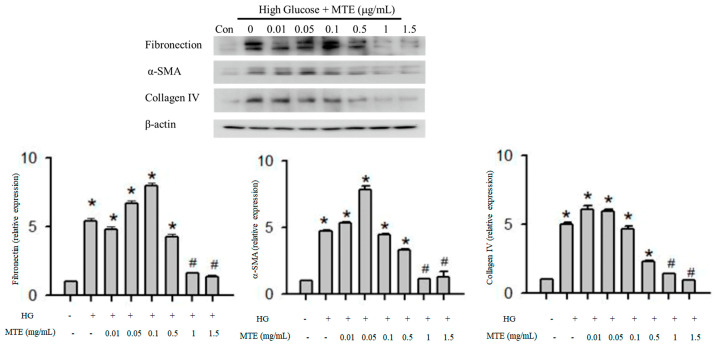
MTE mitigated the expression of fibrosis-related proteins in diabetic mice. The mouse renal mesangial cells were treated with 0, 0.01, 0.05, 0.1, 0.5, and 1 and μg/mL MTE in the presence of high glucose for 48 h. Cells in the absence of high glucose were used as a negative control (Con). Proteins were extracted and subjected to Western blot analysis (upper panel). b-actin was used as a loading control. Lower panel: the data represent the means and standard error obtained from at least three independent experiments. *: *p* < 0.05 compared to the control group. #: *p* < 0.05 compared to the db/db group.

To determine whether MTE influences the expression of fibrosis-related proteins, we conducted Western blot analysis. The expression of fibronectin, α-smooth muscle actin, and collagen IV increased nearly five-fold in response to high glucose treatment. A low dose of MTE (below 0.5 ng/mL) did not affect high glucose-induced fibrosis-related protein expression whereas a high dose of MTE (1 and 1.5 μg/mL) reversed the high glucose-induced phenomenon (Figure 2).

### 2.2. Effects of MTE on Renal Fibrosis In Vivo

To further investigate the effects of MTE on diabetes-induced renal fibrosis, we developed animal models. The body weights and HbA1C levels of the db/db knockout mice with or without MTE were significantly higher than those of the db/m control mice. Interestingly, the albumin loss significantly increased to 0.368 in the db/db mice compared with that (0.010) in the db/m mice. Administration of 50 or 100 mg/kg MTE attenuated the albumin loss to 0.144 and 0.198, respectively. The same is also true for the kidney weight. No overt alterations were found in kidney weight/body weight and total protein loss (Table 1).

**Figure 3:**
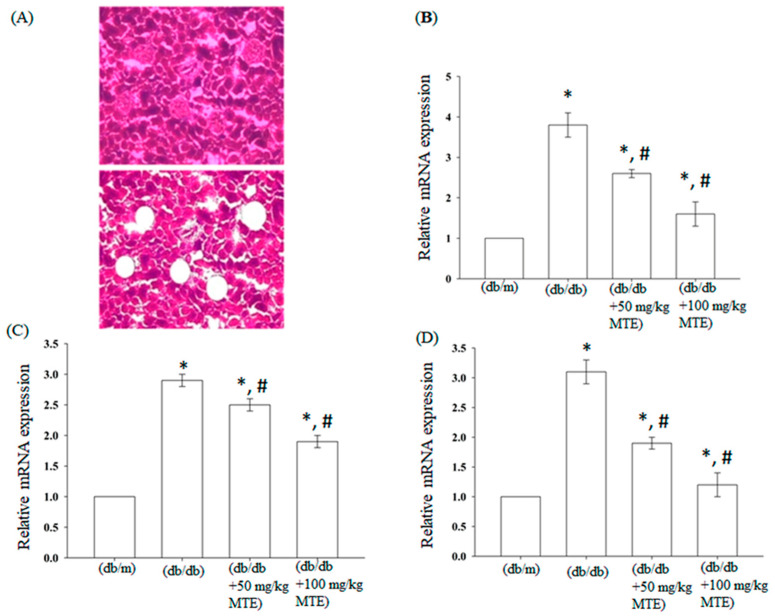
MTE reversed the diabetes-induced expression of fibrosis-related genes. (**A**) Renal tissue sections before (upper panel) and after (lower panel) laser capture microdissection. Quantitative RT-PCR analysis of (**B**) fibronectin, (**C**) TGF-β, and (**D**) collagen IV from the indicated treatment. The data represent the means and standard error obtained from at least three independent experiments *: *p* < 0.05 compared with db/m mice. #: *p* < 0.05 compared with db/db mice without MTE.

To validate whether MTE affects the expression of fibrosis-related genes, we performed quantitative RT-PCR analysis. As shown in Figure 3, the mRNA levels of fibronectin significantly increased by nearly four-folds in the db/db mice, whereas administration of MTE (50 or 100 mg/kg) reduced gene expression. Similarly, administration of MTE also repressed the expression of TGF-b and collagen IV in the db/db mice.

**Figure 4:**
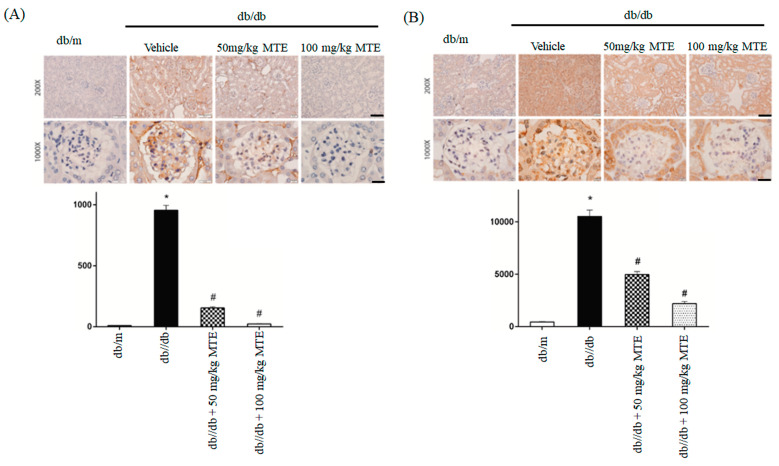
MTE altered the expression of fibrosis-related proteins in diabetic mice. Immunohistochemical staining using anti-fibronectin (**A**) and collagen IV (**B**) antibodies of glomerulus derived from the dd/db mice administered with 0, 50, or 100 mg/kg MTE. db/m mice were used as a negative control. The lower panel represents the means and standard deviation of IDO obtained from six mice. *: *p* < 0.005 compared with db/m mice. #: *p* < 0.005 compared with db/db mice without MTE. Scale bar: 100 μm at 100× and 20 μm at 1000×.

Immunohistochemical staining for collagen IV and fibronectin was conducted to verify the effect of MTE on the expression of fibrosis-related proteins. As shown in Figure 4, the expression of fibronectin and collagen IV was significantly enhanced in the db/db mice. Administration of 50 and 100 mg/kg MTE obviously mitigated the increase in the expression of fibronectin and collagen IV in the db/db mice.

**Figure 5:**
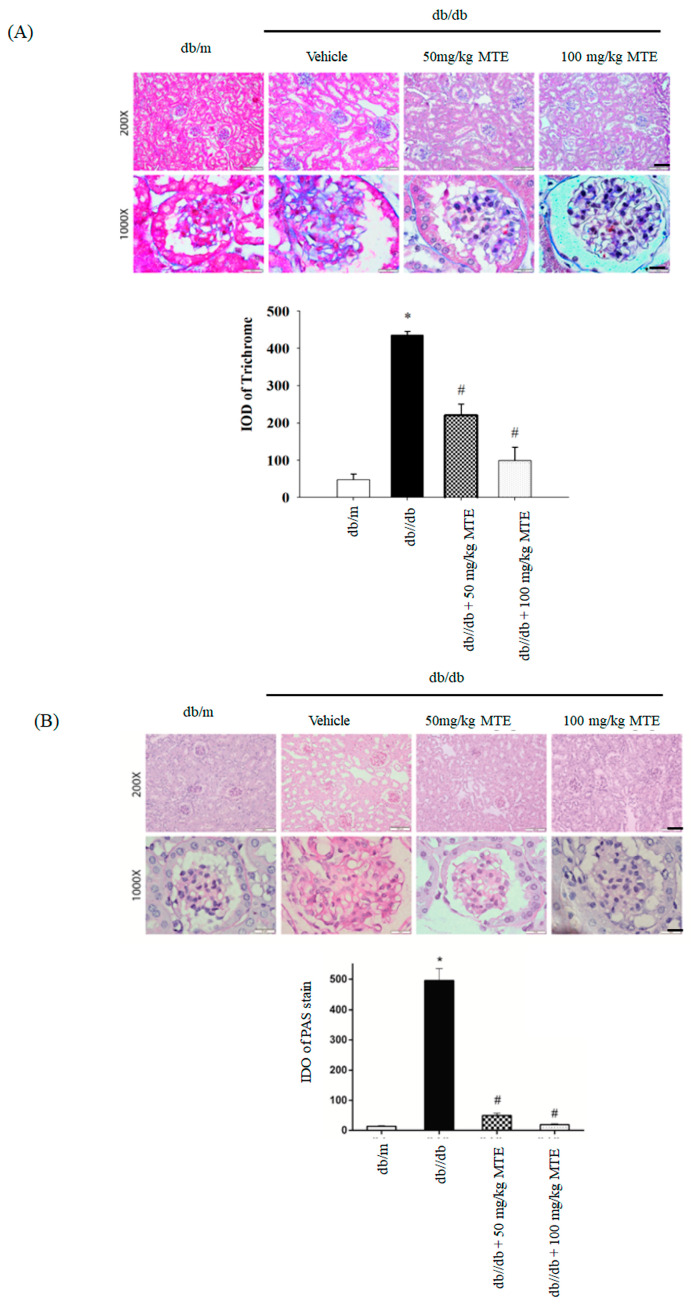
MTE reduced renal fibrosis in the diabetic mice. Trichrome (**A**) and PAS (**B**) staining of glomerulus derived from dd/db mice given 0, 50, or 100 mg/kg MTE. The db/m group was used as a negative control. (B) The lower panel represents the means and standard deviation of IDO obtained from six mice. *: *p* < 0.005 compared with the db/m mice. #: *p* < 0.005 compared with the db/db mice without MTE. Scale bar: 100 μm at 100× and 20 μm at 1000×.

To further determine whether MTE reverses renal fibrosis in db/db mice, we performed trichrome and PAS staining. Increased trichrome staining intensity was found in db/db mice, whereas administration of MTE reversed the phenomenon (Figure 5A). Elevated PAS staining intensity was observed in db/db mice, whereas MTE dose-dependently decreased the PAS signal (Figure 5B).

## 3. Discussion

Diabetes is a major metabolic disease and its prevalence is set to increase annually from 10% in 2021 to an estimated 12.2% in 2045 [23]. DN, one of the complications of diabetes, is the major risk factor for causing end-stage kidney disease [24]. In the progression of DN, hyperglycemia promotes the generation of reactive oxygen species (ROS), triggers inflammation, accumulates ECM, and eventually induces renal fibrosis and renal dysfunction [25]. Flavonoids extracted from plants have received great attention because of their anti-diabetes properties [5]. In this study, we demonstrated the effects of MTE from *M. tanarius* on diabetic nephropathy.

Hyperglycemia triggers damage to podocytes, leading to albumin excretion and DN progression [26]. In the present study, the administration of MTE significantly reduced albumin loss in diabetic mice. In line with our observations, several reports have indicated that flavonoids ameliorate diabetes-induced renal albumin loss. Treatment with tangeretin reduced hyperglycemia-induced renal podocyte injury and recovered renal albumin excretion in db/db mice [27]. In STZ-induced diabetic mice, the levels of serum creatinine and urine increased, whereas administration of Andrographis paniculata extract reversed the STZ-induced phenomenon [28]. Li et al. showed that grape seed proanthocyanidin extracts (GSPE) significantly reduced urine albumin excretion and serum creatinine levels in STZ-induced diabetic mice [29]. Administration of a water extract of Hydrangea paniculate significantly improved the renal function of diabetic mice as evidenced by increased creatine clearance and decreased urine albumin loss [30]. Widyarini et al. demonstrated that Etlingera elatior ethanol extract reduced blood glucose levels and protected renal function by decreasing urine albumin secretion in STZ-induced diabetic mice [31]. Overall, our results indicate that MTE reversal of albumin loss in diabetic mice may ameliorate podocyte injury and block DN progression.

The main pathological characteristic of DN is renal fibrosis caused by renal interstitial inflammation, fibroblast proliferation, and excessive extracellular matrix (ECM) deposition [25]. Fibronectin, α-smooth muscle actin (α-SMA), and collagen IV, which are the three major compounds of ECM, participate in renal fibrosis progression [32,33]. Plant extracts suppress the expression of fibrosis-related proteins and ameliorated DN. Yang et al. demonstrated that a Fufang Zhenzhu Tiaozhi capsule (FTZ), which is composed of eight Chinese traditional herbs, dose-dependently inhibited diabetic-induced fibronectin and collagen IV expression [34]. A Huangkui capsule combined with metformin repressed high glucose-induced tumor growth factor β-1 (TGF-β1) and fibronectin expression in in vitro and in vivo experiments [17]. Xu et al. showed that RTT mitigated renal fibrosis-related protein expression through downregulation of SMYD2 (30%), H3K36me3, and H3K4me3 (53% and 75%, receptively) expression [11]. An extract of Polygala fallax Hemsl (EPF) reduced IL-1β, TNF–α, fibronectin, and collagen IV expression in high glucose-treated human glomerular mesangial cells which suggests that the compounds attenuated DN progression [35]. Moreover, EPF also mitigated the expression of matrix metalloproteinase -2 and -9 [35]. In db/db diabetic mice, administration of 10 and 50 mg/kg Polygoni avicularis–ethanol soluble fraction (ER-PA) significantly reduced inflammation cytokines, TGF, and collagen IV expression [36]. In this study, MTE dose-dependently reduced fibronectin, α-SMA, and collagen IV in renal mesangial cells in the presence of high glucose. Quantitative RT-PCR and immunohistochemical staining analysis also revealed that MTE obviously attenuated fibrosis-related protein expression in db/db diabetic mice. Moreover, PAS and trichrome staining indicated that MTE repressed renal fibrosis in db/db diabetic mice. Collectedly, our results show that MTE exhibited anti-renal fibrosis in diabetic mice.

## 4. Materials and Methods

### 4.1. Sample Preparation

The study materials were provided by NatureWise Biotech & Medicals Corporation (Taipei, Taiwan). Powdered dry leaves of *M. tanarius* (1.5 kg) were immersed in acetone (4.5 L) at room temperature. After filtering, the filtrate was concentrated under reduced pressure to obtain the acetone extract. The extract was resuspended in acetone, mixed with active charcoal powder, filtered by celite 545 on a Büchner funnel, and eluted with aqueous MeOH solution. The 90% MeOH layer was collected and separated by Sephadex LH-20 gel eluted with MeOH to obtain the prenylflavonoid-enriched fraction, which was purified by low-pressure Daiso SP-120-ODS-BP gel (40–60 μm) column to obtain the prenylflavonoids-enriched component. The major compounds of the prenylflavonoid-enriched fraction contained Propolin C (PPC), Propolin D (PPD), and Propolin G (PPG). The recovery rate of prenylflavonoid-enriched component from 1.5 kg dry *M. tanarius* leaves was about 2.63%. The component was finally dissolved in 50% propylene glycol aqueous solution for activity studies. We used a 1:1000 dilution to treat the cells, so the final concentration of propylene glycol was 0.5% in the culture medium.

### 4.2. Cell Culture

The mouse renal mesangial cells were purchased from the American Type Culture Collection (ATCC) and maintained in Dulbecco’s modified Eagle’s medium (DMEM)/F12 medium containing 5% fetal bovine serum. In the control group, the cells were treated with 5.5 mM D-glucose. In the high-glucose group, the cells were stimulated with 25 mM D-glucose for 24 or 48 h. All of the cells were cultured at 37 °C with 5% carbon dioxide.

### 4.3. Quantitative Reverse-Transcription Polymerase Chain Reaction (RT-PCR)

Total RNAs of glomerular mesangium obtained through a VERITAS™ laser-captured dissection system were extracted by TRI Reagent. In brief, 1 μg of total RNA was subjected to first cDNA synthesis and a quantitative polymerase chain reaction [37]. The PCR conditions and primer sequences have also been described in a previous report [37].

### 4.4. Western Blot Analysis

The cells were treated with 0, 0.01, 0.05, 0.1, 0.5, and 1 mg/mL MTE in the presence of 25 mM glucose for 48 h. Proteins were extracted by RIPA buffer. About 30 g of proteins were separated by 10% sodium dodecyl sulfate–polyacrylamide gel and transferred into a nitrocellulose membrane. The membranes were blocked by phosphate buffer saline (PBS) containing 5% non-fat milk and incubated in the indicated antibody at 4 °C overnight. The membranes were incubated with an anti-mouse or anti-rabbit second antibody conjugated with horseradish peroxidase for 1 h at room temperature. Signals were detected by an enhanced chemiluminescent kit.

### 4.5. Animal Model

Eighteen 6-week-old db/db mice (C57BLKS/J Iar-+Leprdb/+Leprdb) were obtained from Jackson Laboratory and randomly divided into three groups: (1) 0 MTE; (2) 50 mg/kg MTE; and (3) 100 mg/kg MTE for 8 weeks (five times per week). Six age-matched db/m mice were used as the negative control group. At the end of the experiment, their body weight, hemoglobin A1C (HbA1C), urine albumin, and urine total protein were measured as described in a previous report [38]. The mice were sacrificed, and their kidneys were immediately removed and weighed. All of the protocols of the animal experiments were certified by the Institutional Animal Care and Use Committee of the Chang Gung Memorial Hospital.

### 4.6. Histological Staining and Immunohistochemical Analysis

The 4 μm paraffin-embedded renal tissue sections were subjected to deparaffinization and rehydration. Three percent H2O2 was used to remove endogenous peroxidase activity. The antigen was retrieved by boiling in 10 mM of citrate buffer (pH 6.0) for 20 min. The sections were incubated with the indicated antibody, and positive signals were detected using a horseradish peroxidase-3-3-diaminobenzidene kit (BioGenex, San Ramon, CA, USA). Hematoxylin staining was conducted as counterstaining.

Masson’s trichrome and periodic acid–Schiff (PAS) staining were performed to analyze collagen deposition and mesangial matrix expansion [15,17]. The quantitative integrated optical density (IOD) was measured as described in a previous report [15].

### 4.7. Statistical Analysis

All data were presented by means ± standard error obtained from at least three experiments. Significance was measured by the Student’s *t*-test using SPSS version 15. A *p*-value < 0.05 was considered significantly different.

## 5. Conclusions

In the presented study, we demonstrated that MTE mitigated high glucose-induced fibronectin, a-SMA, and collagen IV expression in cultured renal mesangial cells. Similarly, our results showed that MTE attenuated the expression of fibrosis-related proteins in a db/db diabetic mouse model. In addition, MTE significantly suppressed the excretion of renal albumin. These findings suggested that MTE negatively regulated DN and may be a potential agent for ameliorating DN progression.

## Figures and Tables

**Table 1:** Effect of PPG on HbA1C, body weight, kidney weight, albumin loss, and total protein loss in diabetic rats.

	db/m	db/db		
	(n = 6)	0 MTE (n = 6)	50 mg/kg MTE (n = 6)	100 mg/kg MTE (n = 6)
HbA1C	4.12 ± 0.16	10.38 ± 0.79 *	9 ± 3.64 *	9.12 ± 0.89 *
Kidney weight	0.30 ± 0.03	0.42 ± 0.03 *	0.36 ± 0.15 *^,#^	0.38 ± 0.03 *^,#^
Body weight	32.56 ± 1.70	49.34 ± 4.66 *	50.65 ± 7.02 *	48.44 ± 4.42 *
Kidney weight/body weight	0.93 ± 0.10	0.86 ± 0.09	0.72 ± 0.30	0.80 ± 0.11
Albumin loss	0.0097 ± 0.0033	0.3685 ± 0.2393 *	0.1436 ± 0.2514 *^,#^	0.1981 ± 0.1227 *^,#^

*: *p* < 0.05 compared to the db/m group. #: *p* < 0.05 compared to the db/db without MTE treatment group.

## Data Availability

Not applicable.
